# Carbon dots with molecular fluorescence and their application as a “turn-off” fluorescent probe for ferricyanide detection

**DOI:** 10.1038/s41598-019-47168-7

**Published:** 2019-07-24

**Authors:** Tianshu Wang, Ailin Wang, Ruixue Wang, Zhaoyang Liu, Ying Sun, Guiye Shan, Yanwei Chen, Yichun Liu

**Affiliations:** Center for Advanced Optoelectronic Functional Materials Research, Key Laboratory for UV Light-Emitting Materials and Technology of the Ministry of Education, Northeast Normal University, Changchun, 130024 People’s Republic of China

**Keywords:** Quantum dots, Nanoparticles

## Abstract

Highly fluorescent carbon dots (CDs) exhibiting molecular fluorescence were synthesized and successfully used for sensing ferricyanide based on fluorescence quenching. We conducted dialysis to purify the CDs and found that the dialysate is also fluorescent. From the mass spectra and quantum yield analyses of the dialysate, it is demonstrated that molecular fluorophores were also synthesized during the synthesis of CDs. By the comparison of fluorescence spectra between CDs and dialysate, it is established that the fluorescence emission of CDs partly originates from fluorophores that are attached to CDs’ surface. The fluorescence quenching caused by ferricyanide is proved to be the overlap of absorption spectra between ferricyanide and CDs. The changes of the absorbance and fluorescence spectra are combined to enhance the detection sensitivity, and the limit of detection is calculated to be 1.7 μM. A good linear response of fluorescence-absorbance combined sensing toward ferricyanide is achieved in the range of 5–100 µM. This method is highly selective to ferricyanide among other common cations and anions, and it is also successfully applied in detecting ferricyanide in real water samples.

## Introduction

Carbon dots (CDs), as carbon-based fluorescent nanomaterials, have attracted extensive attention due to their outstanding properties such as facile synthesis, low toxicity and high quantum yield since the discovery in 2004^1^. CDs are applied in many fields, including bioimaging, mimic enzyme catalyst, white light emission, cancer treatment, supercapacitor, etc^2–7^. There are generally two routes to prepare CDs, namely top-down synthesis of fragmenting or exfoliating large carbon structures such as graphite^8–10^ and bottom-up synthesis of the carbonization of small organic molecules^11,12^. Although the synthesis methods and applications of CDs are plentiful, the fluorescence origin of CDs is still unclear. It is generally conceived that the fluorescence of most CDs originates from the n-π* transitions of the surface defect states^2,13,14^, while some studies revealed the contribution of molecular fluorophores to the fluorescence of CDs^15–17^. In 2011, Giannelis *et al*. discovered the existence of fluorophores in the synthesis process of CDs^15^. Yang’s group separated fluorophores by purifying the CDs product with column chromatography, and demonstrated that the molecular fluorophores led to the fluorescence of CDs^16^. Schneider *et al*. pointed out the influence of fluorophores on the fluorescence of CDs by comparing the similarity of the absorbance and fluorescence spectra between commercially available citrazinic acid and citric acid-based CDs^17^. The contribution of fluorophores to fluorescence emission of citric acid-based CDs has gradually been acknowledged.

The properties of CDs such as highly fluorescent, environment-friendly and facile synthesis make them suitable materials for ion detection. Based on the fluorescence quenching or enhancement caused by the interactions with ions, CDs can be applied in the detection of multiple ions, such as Fe^3+^, Pb^2+^ Hg^2+^, Cr^6+^, Cr^3+^, Be^2+^, ClO^−^, ONOO^−^ and I^−^ ^18–26^. However, detection of ferricyanide ([Fe(CN)_6_]^3−^) by fluorescent probe based on CDs has not been reported previously. Ferricyanide exists in the wastewater of multiple industries. In the gold cyanidation process, the lixiviant CN^−^ is applied to extract gold from low grade ores which usually contain iron minerals. Fe^3+^ reacts with CN^−^ and forms ferricyanide which can be discharged in wastewater^27^. In addition, the wastewater containing ferricyanide was produced in munitions and photographic processing industries. Though the toxicity of ferricyanide is relatively low (LD_50_ = 2970 mg/kg), highly toxic CN^−^ can be released if the coordination centre Fe^3+^ binds to other chelators such as humid acid or if ferricyanide is decomposed by ultraviolet light^28^. Therefore, it is essential to explore practical ferricyanide detection methods. There are a few published methods in ferricyanide detection, but their results are not satisfactory. Some detecting methods are qualitative and involve toxic or radiative elements^29,30^, and the electrochemical determination requires complex fabrication of electrodes^31^. Therefore, exploring green and feasible fluorescent CDs probe for ferricyanide detection is in great demand.

Herein, CDs that exhibit a good sensitivity and selectivity for ferricyanide ions were prepared by a facile one-step microwave-assisted synthesis using citric acid and urea as precursors. After dialyzing the product, the fluorescence behaviour of CDs and dialysate were systematically studied. It is found that molecular fluorophores were also synthesized, and they led to the fluorescence emission of CDs. In the presence of ferricyanide, the fluorescence of CDs at 520 nm is dramatically quenched. The fluorescence quenching is induced by the competition between ferricyanide and CDs in absorbing excitation light. Combining both the changes of absorbance and fluorescence spectra, ferricyanide can be sensitively detected. The method is also successfully applied in detecting ferricyanide in tap water and lake water and has good recovery.

## Results and Discussion

### Characterizations of CDs

**Figure 1:**
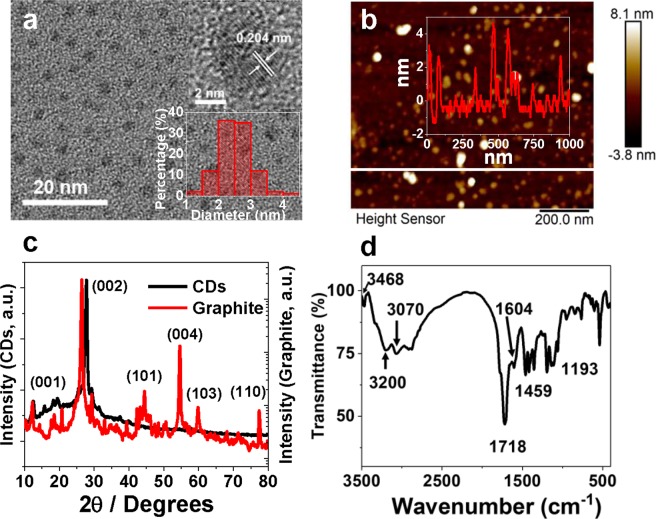
The TEM image of CDs (**a**, inset at top-right: HRTEM image of an individual CD, inset at bottom-left: size distribution of CDs based on 100 counts from the TEM image); the AFM image of CDs (**b**, inset: height profile measured at the white line), XRD pattern of CDs compared with graphite powder (**c**), and FTIR spectrum of CDs (**d)**.

The CDs were successfully synthesized via microwave assisted heating of CA and urea mixture solution. The morphology of CDs was characterized with transmission electron microscopy (TEM) to be isolated quasi-spheres, as shown in Fig. 1a. The inset (bottom-right) of Fig. 1a shows the size distribution of CDs derived from the TEM image. The size of CDs is quite uniform with distribution range 1.1–4.1 nm, and the average diameter of CDs is calculated to be 2.4 nm. The average height of CDs is measured by atomic force microscopy to be 2.9 nm (Fig. 1b), which is consistent with the TEM result. The high-resolution transmission electron microscopy (HRTEM) reveals the existence of lattice structure in CDs (top-right inset of Fig. 1a). The lattice spacing is measured to be 0.204 nm, corresponding to the (101) plane of graphite. The X-ray diffraction (XRD) pattern of CDs shows a sharp peak at 27.8° (Fig. 1c), which corresponds to the (002) plane of graphite. Therefore, it can be deduced that CDs possess graphite crystalline structure. From the fact that CDs exhibit less diffraction peaks than the graphite powder, it can be deduced that the crystal structure of CDs is less well-defined and contains defects. The Raman spectrum of CDs (Supplementary Fig. S1) shows two broad peaks at 1306 (D-band) and 1595 cm^−1^ (G-band), also demonstrating the existence of the disordered carbon and crystalline carbon in CDs respectively^15,32^. Figure 1d shows the FTIR spectrum of CDs. The peaks at 3200, 3070 and 1193 cm^−1^ are assigned to stretching vibrations of -OH, =C-H and C-O, separately. The peaks at 3468 cm^−1^ and 1718 cm^−1^ can be ascribed to the stretching vibrations of N-H and C=O bonds, respectively, confirming the existence of amide functional groups on the surface of CDs. The peaks at 1604 cm^−1^ and 1459 cm^−1^ are characteristic peaks of benzene skeleton vibrations^33^, which can be attributed to the presence of isolated sp^2^ clusters^34^.

**Figure 2:**
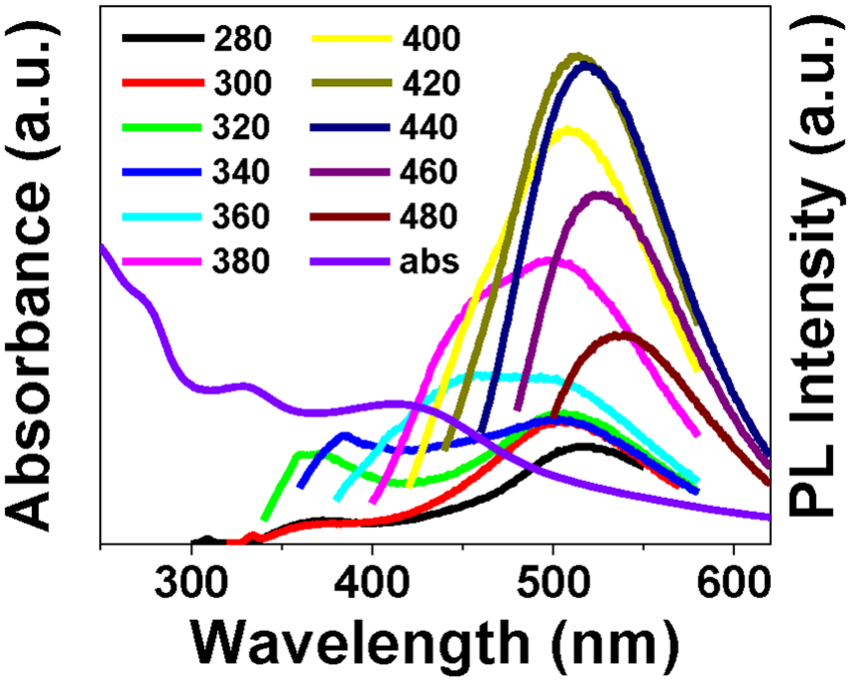
Absorption spectrum and fluorescence emission spectra under different excitation wavelengths of CDs solution.

As shown in Fig. 2, CDs exhibit absorption peaks at 272, 328 and 410 nm. It is worth noticing that our CDs exhibit three emission peaks under different excitation wavelengths. The absorption at 328 nm leads to simultaneous double-peak fluorescence emissions at 370 nm and 520 nm. A plausible explanation is that the reabsorption of the 370 nm light may lead to the emission at 520 nm. There is a shoulder fluorescence peak at 460 nm under 380 nm excitation. The absorption at 410 nm yields the strongest fluorescence signal at 520 nm, with quantum yield 5.3%. The fluorescence at 520 nm is the highest in the original neutral condition (Supplementary Fig. S2a,b), whereas acid and basic environments both quench the fluorescence. The quenching induced by basic environment is more severe and not recoverable after tuning the solution back to neutral (Supplementary Fig. S2c). This phenomenon is probably caused by the structural change of emitting centres, because the absorption at 410 nm of alkali-treated CDs solution didn’t recover after readjusting pH to 7 (Supplementary Fig. S2d).

### Contribution of fluorophores to the emission of CDs

**Figure 3:**
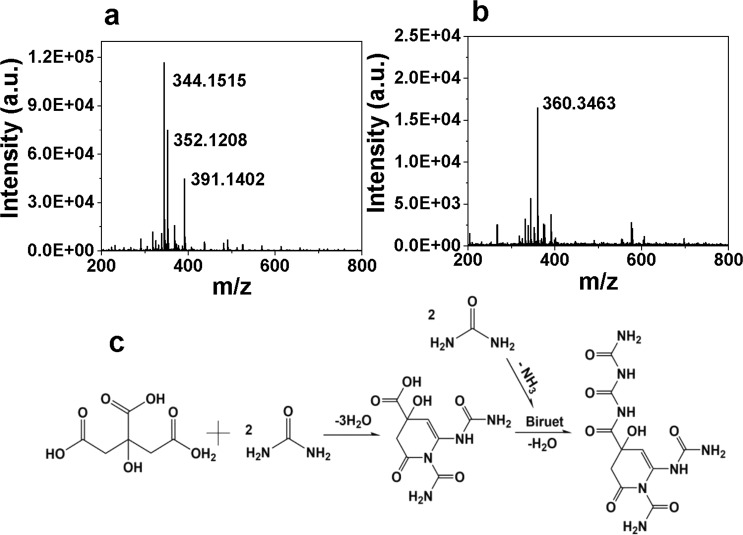
Positive mass spectra of dialysate (**a**) and the dialysate exposed to daylight for a week (**b**). Possible chemical equation for the synthesis of fluorophores (**c**).

CDs were purified with dialysis bag (cut-off molecular weight 500 Da) and it is found that the dialysate is also fluorescent (Supplementary Fig. S3). The quantum yield of dialysate is directly measured to be 25%, which is much higher than the quantum yield of CDs (5.3%). According to the average size estimation from TEM image, the average mass of CDs in Dalton is calculated to be of the order of 1E6 Da (details in Electronic Supplementary Materials). Because it is much higher than the cut-off molecular weight of the dialysis bag, the strong fluorescence of dialysate cannot be attributed to the presence of CDs. Also, no apparent nanoparticle is observed in the TEM image of dialysate (Supplementary Fig. S4). Hence, it can be supposed that fluorophores were also synthesized during the synthesis of CDs. Positive mass spectra (MS) measurement confirms the existence of small molecules in dialysate, and the most prominent peak at 344 m/z suggests a molecular weight of 343 (Fig. 3a). Considering the molecular weight of fluorophores is smaller than the cut-off molecular weight of the dialysis bag, they can pass through the dialysis bag and perform as the fluorescence source of the dialysate. According to the molecular weight derived from MS result, a possible chemical equation of the synthesis of fluorophores is proposed in Fig. 3c, and the name of the fluorophore is *N*^4^-(carbamoylcarbamoyl)-4-hydroxy-2-oxo-6-ureido-3,4-dihydropyridine-1,4(2*H*)-dicarboxamide. However, more purifying work are needed to ascertain the structure of fluorophores.

The CDs solution and dialysate were exposed to daylight for one consecutive week to test their photostability. It is observed that both the absorption peaks at 410 nm and the fluorescence emission peaks at 520 nm of dialysate drop more significantly than those of CDs solution after photobleaching (Supplementary Fig. S5). The fluorescence intensity of CDs remained 71% of its original value after exposure to daylight, in contrast the remaining fluorescence intensity of dialysate is only 11%. It has been found previously that CDs possess higher photochemical stability than fluorophores^2,35–38^, and the susceptibility to photobleaching of dialysate demonstrates its fluorophore essence. The mass spectrum of daylight-exposed dialysate shows a peak at 360 (Fig. 3b), which can be accredited to the oxidation of C=C to epoxide groups. What’s more, the mass spectrum peak intensity of daylight exposed dialysate is an order lower than the dialysate protected from light, indicating that most fluorophores decomposed after the exposure to daylight.

**Figure 4:**
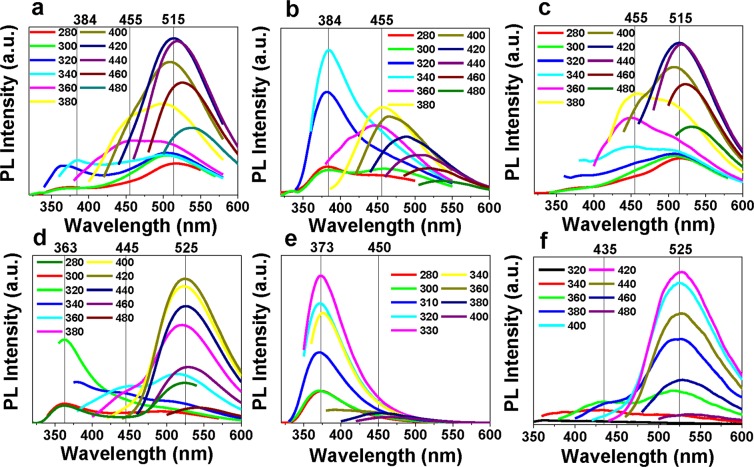
The fluorescence emission spectra of CDs (**a**), reduced CDs (**b**), oxidized CDs (**c**) and dialysate (**d**), reduced dialysate (**e**), oxidized dialysate (**f**) under different excitation wavelengths.

Then, the fluorescence spectra of CDs and dialysate of different surface states were compared to show the contribution of fluorophores in the emission of CDs. CDs and dialysate were treated with reductive NaBH_4_ and oxidative H_2_O_2_ respectively. The untreated CDs and dialysate show all three fluorescence peaks (Fig. 4a,d). After reduction (Fig. 4b,e), both reduced CDs and reduced dialysate show enhanced emission peak at 370 nm, and the emission peak at 520 nm vanishes. The increase of bandgap can be attributed to the enhancement of electron density by reducing carbonyl to hydroxyl groups on the ring of fluorophores^14,39^. After oxidation (Fig. 4c,f), both oxidized CDs and oxidized dialysate show fluorescence peak at 520 nm whereas the fluorescence peak at 370 nm vanishes, as a reverse process of reduction. It is known that the peak position of fluorophores is excitation-independent according to the Kasha’s rule, and the FWHM of fluorescence and absorbance peaks of fluorophores are smaller than CDs due to the presence of multiple lower energy gaps in CDs^17^. Both features are in accordance with our experimental results. As shown in Supplementary Fig. S6, with excitation wavelengths increasing, the fluorescence peak positions around 520 nm of CDs shift more largely than dialysate. Also, The FWHM of emission peaks of dialysate is smaller than CDs, reductive CDs and oxidized CDs (Supplementary Table S1) and the absorbance peak of dialysate is sharper than the CDs solution (Supplementary Fig. S3a), which agrees well with the characteristics of molecular fluorescence.

The absorbance and fluorescence spectra of CDs and dialysate are quite similar, especially the absorbance peaks around 330 nm and 410 nm that corresponds to the fluorescence emissions around 370 nm and 520 nm (Supplementary Table S2). Such similarity vigorously suggests these two fluorescence emissions are closely related to the fluorophores. The change of emission peaks after reduction and oxidation probably arise from the change of fluorophore structures that are destructed or modified by the reduction and oxidation processes. A major difference in fluorescence spectra of CDs and dialysate is that the fluorescence emission peak at 460 nm in CDs (Fig. 4a–c) is more prominent than the counterparts of dialysate (Fig. 4d–f; the direct comparison is given in Supplementary Fig. S7). Because there is a few CDs existing in dialysate, this peak is probably related to the surface states emission of CDs. To gain further understanding of the fluorescence peak at 460 nm, CDs were synthesized with sole CA (c-CDs) and urea (u-CDs) separately. As shown in Supplementary Fig. S8a, u-CDs are not fluorescent, where the sharp drop near the excitation wavelength can be accredited to scattered light; c-CDs exhibit fluorescence spectra which has similar shapes as the fluorescence peak at 460 nm (Supplementary Fig. S8b). It is reported formerly that the emissions of CDs at 370 nm and 520 nm decrease and the emission at 460 nm increases with the increase of CA/urea mass ratio in microwave assisted synthesis of CDs^19^. Therefore, the emissive state at 460 nm can be attributed to the surface defect states of CDs^34^. Because the peak at 460 nm is still retained after redox treatments (Fig. 4b,c), surface modifications by reduction or oxidation have a greater influence on the fluorophores than the surface states of CDs. Therefore, it can be concluded that during the synthesis process, urea and CA react to form fluorophores which accounts for the existence of the fluorescence peaks of CDs at 370 nm and 520 nm, and the fluorescence peak at 460 nm arises from the surface state emission of CDs.

### Sensitive and selective detection of ferricyanide

In our case, ferricyanide causes the quenching of fluorescence emission at 520 nm. Generally, fluorescence quenching can be explained by dynamic quenching or static quenching. The average fluorescence lifetimes of blank CDs and 100 µM ferricyanide spiked CDs solution were measured to be 2.33 and 2.36 ns, respectively (Supplementary Fig. S9). The negligible difference suggests that the luminescence centre is not influenced with the addition of ferricyanide and the quenching mechanism is not dynamic quenching^40^. It is known that the dynamic quenching extent increases whereas static quenching extent decreases with the increase of temperature^40^. The fluorescence quenching extents under 4 °C, 20 °C and 80 °C are compared. Both the original CDs solution and the CDs solution with ferricyanide added shows enhanced fluorescence emissions with the increase of temperature (Supplementary Fig. S10a), but there is no significant change of quenching extents (Supplementary Fig. S10b). Consequently, the fluorescence quenching mechanism is not either static or dynamic quenching. The absorption curves of ferricyanide and CDs solutions were measured, and it is found that the absorbance curve of ferricyanide virtually overlaps with that of CDs (Supplementary Fig. S11). The peak value of CDs is 408 nm, which is quite close to ferricyanide’s peak at 420 nm. Due to the overlapping, ferricyanide compete with CDs in absorbing excitation light quench the fluorescence of CDs. Ferricyanide solution is not fluorescent Therefore, it is verified that in our case competition in absorbing excitation light is the origin of fluorescence quenching. Applications of CDs in ion detection can be further investigated based on absorption spectra overlapping between CDs and the analyte.

**Figure 5:**
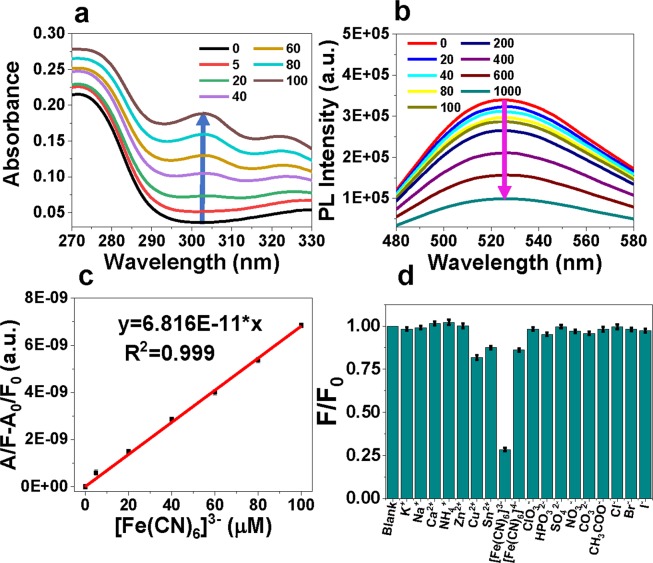
The change of absorption (**a**) and fluorescence (**b**) spectra of CDs solution with different ferricyanide concentrations. The plot of A/F − A_0_/F_0_ versus concentrations of ferricyanide in the range of 0–100 µM (**c**). The quenching of CDs fluorescence in the presence of different ions at concentration 1 mM (**d**).

In most previous studies, ion detection based on CDs depended exclusively on the fluorescence quenching induced by the analytes^18–25,35^. Herein, we present an improved ion detection method, combining both the change of absorption spectra and fluorescence spectra. With the increase of ferricyanide concentration, the characteristic absorption intensity of ferricyanide at 303 nm increases (Fig. 5a) and the fluorescence intensity of CDs at 520 nm decreases (Fig. 5b). A quantity is defined as A/F − A_0_/F_0_, which is the ratio of the absorbance value A at 303 nm and the integrated fluorescence F minus the ratio of the respective blank CDs solutions signals. It is found that there is a linear relationship between A/F − A_0_/F_0_ and the concentration of ferricyanide in the range of 5 μM to 100 μM with a good linear correlation (R^2^ = 0.999) (Fig. 5c). The LOD is calculated to be 1.7 μM (0.35 mg·L^−1^), based on three times of the standard deviation calculated from eight blank samples. The LOD meets the first-class standard of photographic processing industry stipulated by the Integrated Wastewater Discharge Standard of China (GB 8978–1996).

To confirm the selectivity of the as-synthesized CDs to ferricyanide, the fluorescence quenching induced by ten cations and eleven anions on CDs were tested, including Na^+^, K^+^, Ca^2+^, Zn^2+^, Sn^2+^, Cu^2+^, NH_4_^+^, NO_3_^−^, Cl^−^_,_ Br^−^, I^−^, SO_4_^2−^, ClO_3_^−^, HPO_3_^2−^, CH_3_COO^−^, CO_3_^2−^ and [Fe(CN)_3_]^4−^. The concentration of each ion was 1 mM. As shown in Fig. 5d, fluorescence quenching caused by ferricyanide is the most prominent, while some other ions can also effectuate weakened fluorescence quenching. A plausible explanation is that those non-fluorescent ions will compete with CDs in absorbing the incident light due to the partial overlaps in absorption spectra, thus quenching the fluorescence (Supplementary Fig. S11). Also, the presence of other common ions had little interference to the detection of ferricyanide (Supplementary Fig. S12). The high selectivity towards ferricyanide demonstrates the method’s suitability for practical use.

### Ferricyanide detection in real water samples

For practical applications, ferricyanide was spiked and detected in tap water and lake water with concentrations 0, 40 and 80 µM. As shown in Table 1, ferricyanide can also be detected in tap water and lake water with recovery greater than 90% and RSDs less than 5.3% (n = 3). The result confirms that our method is competent to be applied in natural situations.

**Table 1:** Determination of ferricyanide concentration (µM) in real water samples. Three samples were prepared in each test.

Samples	Spiked	Measured	Recovery (%)	RSD, n = 3 (%)
Tap water	0	6.6	—	2.9
	40	44.4	94.1	5.3
	80	82.1	94.5	2.3
Lake water	0	4.9	—	2.4
	40	42.8	95.0	1.6
	80	80.5	94.6	1.3

## Discussion (Conclusion)

Carbon dots with CA and urea as raw materials were synthesized via microwave assisted method. From quantum yield measurements and the mass spectra of the dialysate of CDs, it is demonstrated that small fluorophores were also synthesized in the synthesis of CDs. By the comparison of fluorescence spectra between CDs and dialysate in different surface states, it is established that fluorophores attach on the surface of carbon dots and the fluorescence emissions of CDs can be attributed to the surface defect states and fluorophores emissions. Further works are needed in purifying the fluorescent molecules and elucidating the exact structure of fluorophores. Based on the combination of absorbance spectra change and fluorescence quenching of CDs, the as-synthesized CDs can be applied in the detection of ferricyanide with good sensitivity and selectivity. The successful detection of ferricyanide in real water samples demonstrates the practicability of our detection method.

## Methods

### Instruments

The morphology and size of the sample were characterized by a transmission electron microscopy (FEI/Philips CM200 LaB6 microscope) manipulating at 200 kV and an atomic force microscopy (Dimension IconTM). UV-vis absorption spectra were recorded on a UV-visible spectrophotometer (Shimadzu UV 2600). Fluorescence spectra and quantum yields were recorded on a fluorescence spectrophotometer (PTI-Quanta Master 400). Fluorescence lifetimes were recorded on a fluorescence spectrophotometer (PTI-Quanta Master 8000). The Raman spectrum was collected by a Raman spectrometer (HORIBA JOBIN YVON HR800). X-ray diffraction patterns were recorded with an X-ray diffraction machine (D8 ADVANCE), using Cu Kα radiation (λ = 1.5406 Å). Fourier transform infrared (FTIR) spectra were collected on a FTIR spectrometer (Nicolet is-10). Positive mass spectra were recorded on a liquid chromatography–mass spectrometry system (microtof II).

### Synthesis, photobleaching and surface modification of CDs

CDs were synthesized by a microwave-assisted method^19^. Briefly, 1 g of citric acid monohydrate (CA) and 3 g of urea were dissolved in 15 mL of water. Then, the solution was heated in a domestic microwave oven (700 W) for 5 min, obtaining black solid. The solid was dissolved in 60 mL of water, centrifuged (12000 rpm, 10 min) and filtered through 0.22 µm polyethersulfone membrane to remove large particles. Finally, the solution was dialyzed with dialysis bag (cut off molecular weight 500 Da) against water for a week, obtaining CDs solution (inside the dialysis bag) and dialysate (outside the dialysis bag).

In the photobleaching test, CDs solution and dialysate were placed on a windowsill of an east facing window in the authors’ laboratory for a consecutive week, mimicking natural photobleaching environment. In control group, CDs solution and dialysate were kept in dark environment.

The CDs solution was treated with reductive NaBH_4_ and oxidative H_2_O_2_ to modify the surface. 1 mL of CDs (1 mg·mL^−1^) solution was diluted to 10 mL and 0.3 g of NaBH_4_ was added to it, stirring for 24 h; 1 mL of CDs solution (1 mg·mL^−1^) was mixed with 1 mL of 30% H_2_O_2_, protecting from light for 24 h, then diluted with water to 10 mL. Dialyses were conducted to remove the reductive and oxidative agents, obtaining the reduced CDs, and oxidized CDs. The dialysate was treated with the above procedure without dialysis, obtaining reduced dialysate and oxidized dialysate.

Carbon dots were also synthesized from sole CA and urea. 1 g of CA and 3 g of urea were respectively dissolved in 15 mL water, heated by the above procedure, obtaining viscous yellowish fluid from CA (c-CDs) and white solid from urea (u-CDs).

### Quantum yield measurement

The quantum yields of CDs and dialysate were directly measured with the fluorescence spectrophotometer in integration sphere mode, based on the formula below:

1 $${\rm{quantum}}\,{\rm{yield}}=\frac{{\rm{photons}}\,{\rm{emitted}}}{{\rm{photons}}\,{\rm{absorbed}}}$$

The photons absorbed were the integrated fluorescence value of water minus the sample’s in the excitation range 380–450 nm, and the photons emitted were the integrated fluorescence value of the sample minus water’s in the fluorescent emission range 450–700 nm (Supplementary Fig. S13). The excitation wavelength was 420 nm.

### Ferricyanide detection

In the selectivity test, 0.1 mL of CDs solution (10 mg·mL^−1^) and solutions of different ions (final concentration 1 mM) was added to a series of volumetric flasks, and the final volumes were adjusted to 10 mL with water. For each ion, three samples were prepared for measurements. After 15 min of equilibration, fluorescence and absorbance spectra tests were conducted. In the sensitivity test, CDs solutions with different concentrations of ferricyanide were prepared and equilibrated as the above procedure. The fluorescence emission range 480–580 nm under 420 nm excitation was used to calculate the integrated fluorescence intensity F. The characteristic absorbance A of ferricyanide at 303 nm was calculated by averaging the values of absorbance in the range of 300–305 nm. The detection limit was calculated using the following equation:

2 $${\rm{LOD}}=\frac{3\cdot s}{k}$$

where *s* is the standard deviation of eight blank signals, and *k* is the slope of the calibration line.

### Real water sample tests

To evaluate the applicability of our method, different concentrations of ferricyanide in real water samples were measured. Tap water in the authors’ laboratory was used, and lake water was collected in the South Lake of Changchun, China. Both samples were filtered with 0.22 µm polyethersulfone membrane to remove large particles. Ferricyanide solutions with final concentrations 40 and 80 µM were spiked in real water samples for the recovery tests.

## Supplementary information

Electronic Supplementary Material

## Data Availability

The datasets generated during and/or analysed during the current study are available from the corresponding author on reasonable request.
