# Supplementary material for: Carbon dots with molecular fluorescence and their application as a “turn-off” fluorescent probe for ferricyanide detection
Source: Sci Rep. 2019 Jul 24;9:10723. doi: 10.1038/s41598-019-47168-7 (PMC6656716; doi:10.1038/s41598-019-47168-7)
Supplement: Supplementary file 1 — Electronic Supplementary Material [file 41598_2019_47168_MOESM1_ESM.docx]

**Electronic Supplementary Material**

# Carbon dots with molecular fluorescence and their application as a “turn-off” fluorescent probe for ferricyanide detection

**Tianshu Wang^1^, Ailin Wang^1^, Ruixue Wang^1^, Zhaoyang Liu^1^, Ying Sun^1^, Guiye Shan^1*^, Yanwei Chen^1^, Yichun Liu^1^**

^1^Centre for Advanced Optoelectronic Functional Materials Research, Key Laboratory for UV Light-Emitting Materials and Technology of the Ministry of Education, Northeast Normal University, Changchun, People’s Republic of China,130024

* Corresponding author.

Tel.: (86) 431-85099803; (86) 431-85099748

E-mail: [shangy229@nenu.edu.cn](mailto:shangy229@nenu.edu.cn)

## Estimation of the molecular weight of CDs

The molecular weight of CDs in the unit of Dalton is calculated with the formula below; it is assumed that CDs are spherical, and the density of CDs is the same as graphite.

$$\begin{aligned} M=N_{A}\frac{4\pi}{3}\rho_{graphite}r^{3} \#\left( 1 \right) \end{aligned}$$

Where

- *M* is the molecular weight of CDs;
- *N_A_* is the Avogadro constant;
- $\rho_{graphite}$ is the density of graphite (2.23 g·cm^-3^);
- *r* is the mean radius of CDs derived from the transmission electron microscopy result.

## Figures

**Fig. S1** Raman spectrum of CDs

**b**

**d**

**c**

**a**

**Fig. S2** Fluorescence spectra (**a**) and integrated fluorescence intensities (**b**) of CDs aqueous solutions at different pH, and the fluorescence (**c**) and absorption (**d**) spectra after adjusting acid and basic CDs aqueous solutions back to neutral (1 recovery and 13 recovery

**a**

**b**

**Fig. S3** The absorbance spectra (**a**) and fluorescence spectra (**b**) of CDs and dialysate under 420 nm excitation


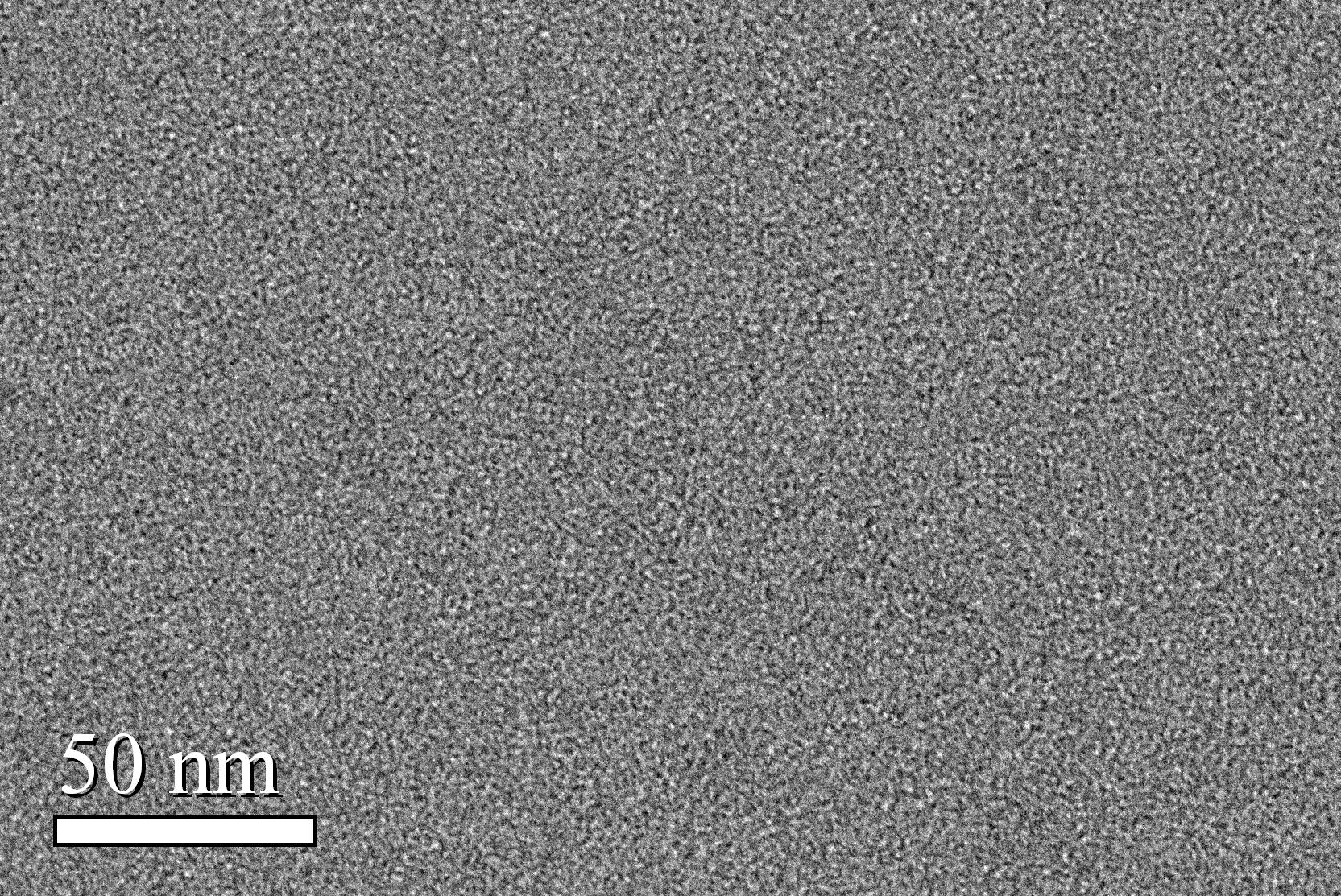


**Fig. S4** The TEM image of dialysate

**a**

**b**

**c**

**d**

**Fig. S5** The change of absorption and fluorescence spectra of CDs (**a, c**) and dialysate (**b, d**) after 1 week’s exposure to daylight

**Fig. S6** Comparison of emission peak positions of dialysate and CDs solution under different excitation wavelengths

**a**

**b**

**c**

**Fig. S7** Fluorescence spectra of untreated (**a**), reduced (**b**) and oxidized (**c**) CDs and dialysate under 360 nm excitation

**a**

**b**

**Fig. S8** Fluorescence spectra of u-CDs (**a**) and c- CDs (**b**) under different excitation wavelengths

**Fig. S9** Fluorescence lifetime decay curves of blank CDs solution and CDs solution with ferricyanide concentration 100 µM

**b**

**a**

**Fig. S10** The PL spectra (**a**) and quenching extent (**b**) of blank CDs and CDs with ferricyanide concentration 100µM at different temperatures

**Fig. S11** Absorption spectra of CDs, K_3_[Fe(CN)_6_], K_4_[Fe(CN)_6_], Cu(NO_3_)_2_ and SnCl_2_ solutions

**Fig. S12** Fluorescence quenching of CDs caused by ferricyanide (0.1 mM) in the presence and absence of other common salts (1 mM)

**Fig. S13** The PL spectra of dialysate, CDs and water under 420 nm excitation in quantum yield measurement

## Tables

**Table S1** Comparison of Full Width at Half Maximum (FWHM) between dialysate and untreated, reduced and oxidized states CDs at different emission peaks

| **FWHM (nm)**  **Emissive peak** | **Dialysate** | **CDs** | **Reduced CDs** | **Oxidized CDs** |
| --- | --- | --- | --- | --- |
| 370 nm | 24 |  | 40 |  |
| 460 nm | 52 | 63 | 66 | 69 |
| 520 nm | 46 | 107 |  | 80 |

**Table S2** Comparison of absorbance and fluorescence peak locations between CDs and dialysate

| Materials    Location (nm) | CDs | Dialysate |
| --- | --- | --- |
| Absorbance peak no. 1 | 330 | 330 |
| Absorbance peak no. 2 | 408 | 409 |
| Fluorescence peak around 370 nm | 363 | 384 |
| Fluorescence peak around 460 nm | 455 | 445 |
| Fluorescence peak around 520 nm | 515 | 525 |
